# Antimicrobial Resistance and Virulence Genes in *Escherichia coli* Isolated from Raptors in Central Italy

**DOI:** 10.3390/vetsci13040342

**Published:** 2026-03-31

**Authors:** Giulia Cagnoli, Fabrizio Bertelloni, Alessia Di Paolo, Renato Ceccherelli, Valentina Virginia Ebani

**Affiliations:** 1 Department of Veterinary Science, University of Pisa, Viale delle Piagge 2, 56124 Pisa, Italy; giulia.cagnoli@phd.unipi.it (G.C.); alessia.dipaolo@studenti.unipi.it (A.D.P.); valentina.virginia.ebani@unipi.it (V.V.E.); 2 LIPU Bird Life Italia, via Pasubio 3 bis, 43122 Parma, Italy; apusvet.cruma@libero.it; 3 Centre for Climate Change Impact, University of Pisa, Via Del Borghetto 80, 56124 Pisa, Italy

**Keywords:** *Escherichia coli*, raptors, EPEC, *ast*A, antimicrobial resistance, resistance genes

## Abstract

Wildlife can serve as a potential reservoir and spreader of resistant and pathogenic bacteria. Raptors, occupying the ecological position of apex or mesopredators, integrate exposure across different habitats and therefore serve as bioindicators of environmental dissemination of pathogens. In this study, we isolated 54 *Escherichia coli* (*E. coli*) strains from feces sampled from 64 raptors admitted to a wildlife rescue center in Central Italy. Phenotypic antimicrobial susceptibility testing was conducted, followed by molecular screening for resistance genes. Additionally, the presence of intestinal *E. coli* pathotypes, including STEC, EHEC, EPEC, ETEC, EAEC, EIEC, and NTEC, was evaluated through virulence gene analysis. Results indicated notable resistance to commonly used antimicrobials, with the highest percentages observed for ampicillin (40.74%), fluoroquinolones (31.48%), and tetracycline (25.93%). Molecular analysis of phenotypically resistant isolates identified the presence of several resistance genes, including *bla_TEM_* (13 isolates), *bla_CTX-M_* (4 isolates), *bla_CMY-2_* (3 isolates), *bla_SHV_* (1 isolate), *tet*(A) (9 isolates), *tet*(B) (4 isolates), *cat1* (1 isolate), and *cmlA* (2 isolates). Furthermore, 29.63% of isolates were classified as multidrug-resistant (MDR) and 7.41% as extensively drug-resistant (XDR). Regarding virulence profiles, one isolate harboring *eaeA*, *escV*, and *ast*A genes was classified as atypical EPEC, while 27.78% isolates had only the *ast*A gene, preventing precise pathotype assignment. These findings highlight the circulation of antimicrobial-resistant and potentially pathogenic *E. coli* strains within raptor populations in Central Italy, emphasizing the zoonotic potential and reaffirming the role of raptors as bioindicators within a One Health approach.

## Simple Summary

Wildlife is increasingly recognized as both a potential reservoir and vector for various pathogens, as well as a valuable bioindicator of environmental health. Raptors, in particular, serve as ecological sentinels due to their position as apex or mesopredators. By preying on small mammals, birds, reptiles, and invertebrates, or scavenging on carcasses and anthropogenic waste, they integrate exposure across multiple trophic levels and habitats. This makes them highly relevant for One Health surveillance initiatives. In this study, we observed that a high proportion of *Escherichia coli* (*E. coli*) isolates from raptors exhibit resistance to critical antimicrobials such as beta-lactams and fluoroquinolones. Furthermore, approximately one-third of these isolates were multidrug-resistant. These findings underscore the role of raptors as bioindicators, reflecting the circulating antimicrobial resistance (AMR) within their ecosystems. Conversely, only a limited number of *E. coli* strains belonging to major gastrointestinal pathogenic types were detected, indicating either limited circulation of these strains or a low prevalence of carriers among the raptor population. This work reinforces the importance of raptors as bioindicators, owing to their behavior and diet. Understanding the prevalence of antimicrobial-resistant and virulent *E. coli* in raptors is not only relevant to veterinary and ecological health but also has direct implications for human health.

## 1. Introduction

*Escherichia coli* (*E. coli*) is an ubiquitous commensal Gram-negative bacterium that inhabits the intestinal tract of humans and animals [1]. While generally harmless, certain strains possess pathogenic potential and can cause diseases in both humans and animals. Several pathogenic strains, known as Intestinal Pathogenic *E. coli* (InPEC), follow the fecal-oral transmission route, leading to various intestinal disorders [2]. InPECs are highly diverse and represent a significant cause of diarrhea in mammals worldwide [3]. Based on the mechanism of pathogenicity, InPEC can be classified into several pathotypes: Enteropathogenic *Escherichia coli* (EPEC), Shiga Toxin-producing *E. coli* (STEC), Enterohemorrhagic *E. coli* (EHEC), Enterotoxigenic *E. coli* (ETEC), Enteroinvasive *E. coli* (EIEC), Enteroaggregative *E. coli* (EAEC), and Necrotoxic *E. coli* (NTEC) [2,4,5,6].

*E. coli* inherently lacks intrinsic resistance to antimicrobials; however, it readily acquires resistance through various mechanisms. This property is largely attributed to its genomic plasticity, capacity to share mobile genetic elements such as plasmids and transposons, and its widespread presence across diverse hosts and environments [7,8]. The primary driver of antimicrobial resistance is the acquisition and accumulation of mobile resistance genes. In recent years, a significant concern has emerged regarding Enterobacteriaceae, particularly *E. coli*, with the rise and dissemination of resistance genes targeting key antibiotic classes [7]. Notably, the spread of beta-lactamase genes such as *bla*_TEM_ and *bla*_CTX-M_ confers resistance to penicillins and cephalosporins, and *bla*_NDM_ and *bla*_OXA_ genes that are associated with carbapenem resistance [9]. The emergence and dissemination of mobile colistin resistance (*mcr*) genes are especially alarming, given that colistin is often considered a last-resort antimicrobial [10]. Additionally, the extensive overuse of tetracycline, especially in the past, has led to the widespread distribution of tetracycline resistance genes (*tet*) among *E. coli* [7].

The most recent surveillance report of the European Food Safety Authority (EFSA) and the European Centre for Disease Prevention and Control (ECDC) on antimicrobial resistance (AMR) in Europe underscores the ongoing rise in resistance among zoonotic and indicator *E. coli* strains, particularly against β-lactams and fluoroquinolones [11]. Moreover, there is an increasing dissemination of strains exhibiting resistance to multiple antimicrobials. The global emergence of multidrug-resistant (MDR) bacteria presents a significant public health challenge, as it severely restricts available therapeutic options [12,13,14,15].

It is widely acknowledged that the health of humans, animals, and the environment is deeply interconnected. From this perspective, wildlife not only serves as reservoirs and vectors for pathogens and antimicrobial-resistant bacteria, but also plays a crucial role as bioindicators of environmental health. By monitoring wildlife populations, we can gain valuable insights into the presence and circulation of contaminants and infectious agents within a given geographic area, thereby reflecting the impacts of human activities on the ecosystem [16,17,18].

Birds, especially synanthropic and semi-synanthropic species, can serve as valuable bioindicators for antimicrobial-resistant and zoonotic microorganisms. Due to their habitats, behaviors, and diets, these birds are frequently exposed to antimicrobials, their residues, and resistant and pathogenic bacteria, making them effective sentinels for monitoring environmental and public health risks [19,20].

Raptors serve as valuable ecological sentinels due to their role as apex or mesopredators. By preying on a diverse range of small mammals, birds, reptiles, and invertebrates, as well as scavenging on carcasses and anthropogenic waste, they integrate exposure across multiple trophic levels and habitat types [21,22,23]. Scientific evidence indicates that carnivorous and omnivorous species are generally at greater risk of harboring antimicrobial-resistant and pathogenic bacteria due to their diets. Additionally, top predators, which forage over extensive geographic areas, face increased risks of exposure and accumulation of these bacteria. Among avian species, raptors and gulls are particularly vulnerable to such risks, given their feeding behaviors and ecological niches [17].

Previous studies have documented the presence of *E. coli* strains in raptors that exhibit resistance to critical antimicrobials used in human therapy, such as cephalosporins or colistin, associated with the acquisition of mobile resistance genes [23,24,25,26]. Additionally, pathogenic *E. coli* have been identified in raptors, indicating their role as potential reservoirs and vectors for the dissemination of virulent strains [27,28]. However, data on the occurrence of AMR and pathogenic *E. coli* in raptors from Italy remain scarce. One investigation in northwest Italy reported that 40% and 13% of *E. coli* isolates from raptors and other wild animals were multidrug-resistant (MDR) and extended-spectrum beta-lactamase (ESBL)-producing. However, this study primarily focused on examining the impact of hospitalization in rescue centers on antimicrobial resistance development and relied solely on phenotypic testing [19]. Another study conducted in central Italy evaluated *E. coli* isolates from raptors and other wild birds admitted to rescue centers, revealing a high prevalence of resistance to ampicillin (85%) and other beta-lactams, as well as ciprofloxacin (18%). ESBL-producing isolates were identified in 8% of samples, and MDR strains accounted for 17%. Similar to the first, this research assessed antimicrobial resistance exclusively through phenotypic methods [29]. In contrast, our working group investigated the presence of STEC-associated genes in the feces of various wild bird species. Using a culture-independent approach, *stx1*, *stx2*, *eaeA*, and *hylA* genes were detected in the intestine of a single little owl [27].

The aim of this study was to isolate and characterize *E. coli* from raptors admitted to a wildlife rescue center in Central Italy. We evaluated antimicrobial resistance using both phenotypic and genotypic approaches, and assessed the presence of virulence factors associated with major InPEC pathotypes through molecular techniques.

## 2. Materials and Methods

### 2.1. Sampling

During 2023 and 2024, a total of 64 fecal samples were collected from raptors (one sample per animal) belonging to 10 different species. All birds were admitted to a wildlife rescue center in Central Italy. The sampled species included 13 Eurasian buzzards (*Buteo buteo*), 10 common kestrels (*Falco tinnunculus*), 10 little owls (*Athene noctua*), 9 peregrine falcons (*Falco peregrinus*), 7 barn owls (*Tyto alba*), 7 tawny owls (*Strix aluco*), 3 scops owls (*Otus scops*), 2 black kites (*Milvus migrans*), 2 sparrowhawks (*Accipiter nisus*), and 1 honey buzzard (*Pernis apivorus*). The raptors were admitted primarily due to trauma and housed individually in single cages. Fecal samples were collected non-invasively from the bottom of each cage, as soon as possible after the birds’ arrival at the rescue center, and only from raptors not yet receiving antibiotic treatments. No ethical approval was required, as no biological material was collected directly from the birds. Each sample was placed in a sterile plastic tube and transferred within 3 h, kept in a cool bag at 4 °C, to the Avian Pathology Laboratories of the Department of Veterinary Sciences, University of Pisa, for bacteriological examination.

### 2.2. Escherichia coli Isolation

Fecal samples were initially pre-enriched in buffered peptone water (BPW; Oxoid Ltd., Basingstoke, UK) at a 1:10 dilution and incubated at 37 °C for 24 h. Following enrichment, a loopful of cultures was streaked onto selective Tryptone Bile X-GLUC (TBX) agar (Biolife, Milan, Italy) and incubated at 42 °C for 24 h. From each TBX plate, a presumptive *E. coli* colony was selected and streaked onto Tryptic Soy Agar (TSA; Biolife) to obtain pure isolates. Confirmation of *E. coli* was achieved through a species-specific PCR targeting the *uspA* gene. The primers used were Up (5′-CCGATACGCTGCCAATCAGT-3′) and Down (5′-ACGCAGACCGTAGGCCAGAT-3′), which amplified an 884 bp fragment of the gene. The PCR was performed with an annealing temperature of 70 °C; *E. coli* ATCC 25922 served as the positive control, while sterile distilled water was used as the negative control in all PCR reactions [30]. Confirmed *E. coli* strains were preserved in Brain–Heart Infusion (BHI) broth (Oxoid Ltd.) supplemented with 30% glycerol and stored at −80 °C for downstream analyses.

### 2.3. Antimicrobial Susceptibility Tests

The antimicrobial susceptibility profile of all *E. coli* isolates was determined using the Kirby–Bauer disk diffusion method [31]. A total of 15 antimicrobials spanning nine different classes were tested. The disks (Oxoid Ltd.) used included: ampicillin (10 µg), amoxicillin–clavulanate (20/10 µg), cefoxitin (30 µg), cefotaxime (30 µg), ceftiofur (30 µg), imipenem (10 µg), ertapenem (10 µg), aztreonam (30 µg), chloramphenicol (30 µg), tetracycline (30 µg), enrofloxacin (5 µg), ciprofloxacin (5 µg), gentamicin (10 µg), amikacin (30 µg), and trimethoprim–sulfamethoxazole (1.25/23.75 µg). Zone diameters were interpreted in accordance with Clinical and Laboratory Standards Institute (CLSI) guidelines [32]. *E. coli* ATCC 25922 was used as a quality control strain in all tests [32].

Colistin resistance was evaluated via the broth microdilution method as recommended by CLSI [33]. Colistin sulfate (CARLO ERBA Reagents, Cornaredo, Italy) was prepared in two-fold serial dilutions ranging from 256 to 0.5 µg/mL in cation-adjusted Mueller–Hinton (MH) broth (Oxoid Ltd.). Isolates with minimum inhibitory concentrations (MIC) > 2 µg/mL were classified as resistant [32]. *E. coli* ATCC 25922 was used as a quality control strain for the colistin MIC test [32].

Based on their antimicrobial resistance patterns, isolates were categorized following the definitions established by Magiorakos et al. [34]. Briefly, strains resistant to at least one antimicrobial in three or more antimicrobial classes were classified as multidrug-resistant (MDR); strains resistant to at least one antimicrobial in all but two or fewer antimicrobial classes were classified as extensively drug-resistant (XDR); and strains resistant to all antimicrobials across all antimicrobial classes were classified as pandrug-resistant (PDR).

### 2.4. Molecular Analyses

DNA was extracted from fresh *E. coli* cultures grown on TSA using the Quick-DNA Miniprep Plus Kit (Zymo Research, Irvine, CA, USA), following the manufacturer’s instructions. PCR assays were conducted on a SimpliAmp™ Thermal Cycler (Applied Biosystems, Waltham, MA, USA). PCR reactions were performed in a total volume of 25 μL, comprising 12.5 μL of DreamTaq Hot Start Master Mix (Life Technologies Italia, Milan, Italy), 0.1 μM of each primer, 3 μL of extracted DNA, and nuclease-free water to reach the final volume. The cycling conditions were as follows: an initial denaturation at 95 °C for 10 min; 35 amplification cycles consisting of denaturation at 95 °C for 1 min, annealing at temperatures specified in Table 1 and Table 2 for 1 min, and extension at 72 °C for 2 min; followed by a final extension step at 72 °C for 10 min. Each PCR reaction included sterile water as a negative control and DNA from previously characterized *E. coli* strains positive for the target genes as positive controls. PCR products were separated by electrophoresis on a 1.5% agarose gel at 100 V for 45 min, with a 100 bp DNA ladder (Solis BioDyne, Tartu, Estonia) serving as a molecular size marker. Gels were stained with ethidium bromide and visualized under UV illumination.

Positive PCR amplicons were purified and submitted to BMR Genomics (Padova, Italy) for bidirectional sequencing to confirm their identity. Sequence data were analyzed using BioEdit [35], and results were compared to online BLAST databases (Accessed on 10 April 2025).

#### 2.4.1. Genotypic Resistance

The isolates exhibiting resistance plus intermediate susceptibility to penicillins (ampicillin and/or amoxicillin-clavulanate) and/or cephalosporins (cefoxitin, cefotaxime, and/or ceftiofur) were subjected to molecular analyses to identify the presence of β-lactamases-encoding genes, including *bla*_TEM_, *bla*_SHV_, and *bla*_CTX-M_. Additionally, these strains were screened for the presence of *bla*_CMY1_ and *bla*_CMY2_ genes, responsible for AmpC β-lactamases production. Strains exhibiting resistance plus intermediate susceptibility to carbapenems (imipenem and/or ertapenem) were tested for carbapenemase genes, including *bla*_NDM_, *bla*_VIM_, *bla*_IMP_, *bla*_KPC_, and *bla*_OXA-48_ genes. Furthermore, isolates resistant or intermediate to chloramphenicol and tetracyclines were analyzed for the presence of *cat1* and *cmI*A genes, and *tet*(A) and *tet*(B) genes, respectively. Primers sequences and PCR conditions are detailed in Table 1.

**Table 1:** Primers and PCR conditions adopted for the detection of the investigated resistance genes.

Target Gene	Primer	Sequence 5′ → 3′	Annealing Temp. (°C)	Amplicon Size (bp)	References
*bla*_TEM_	MultiTSO-T_for	CATTTCCGTGTCGCCCTTATTC	60	800	[36]
	MultiTSO-T_rev	CGTTCATCCATAGTTGCCTGAC			
*bla*_SHV_	SHV-F	TTCGCCTGTGTATTATCTCCCTG	50	854	[37]
	SHV-R	TTAGCGTTGCCAGTGYTCG			
*bla*_CTX-M_	CTX-F	ATGTGCAGYACCAGTAARGTKATGGC	60	593	
	CTX-R	TGGGTRAARTARGTSACCAGAAYCAGCGG			
*bla*_CMY-1_ group	CMY1-F	GTGGTGGATGCCAGCATCC	58	915	
	CMY1-R	GGTCGAGCCGGTCTTGTTGAA			
*bla*_CMY-2_ group	CMY2-F	GCACTTAGCCACCTATACGGCAG	58	758	
	CMY2-R	GCTTTTCAAGAATGCGCCAGG			
*bla*_NDM_	NDM-F	GGTTTGGCGATCTGGTTTTC	52	621	[38]
	NDM-R	CGGAATGGCTCATCACGATC			
*bla*_KPC_	KPC-F	CGTCTAGTTCTGCTGTCTTG	52	798	
	KPC-R	CTTGTCATCCTTGTTAGGCG			
*bla*_OXA-48_	OXA-F	GCGTGGTTAAGGATGAACAC	52	438	
	OXA-R	CATCAAGTTCAACCCAACCG			
*bla*_IMP_	IMP-F	GGAATAGAGTGGCTTAAYTCTC	52	232	
	IMP-R	GGTTTAAYAAAACAACCACC			
*bla*_VIM_	VIM-F	GATGGTGTTTGGTCGCATA	52	390	
	VIM-R	CGAATGCGCAGCACCAG			
*tetA*	tetAF	GCTACATCCTGCTTGCCTTC	64	210	[39]
	tetAR	CATAGATCGCCGTGAAGAGG			
*tetB*	tetBF	TTGGTTAGGGGCAAGTTTTG	64	659	
	tetBR	GTAATGGGCCAATAACACCG			
*cat1*	CATI-F	AGTTGCTCAATGTACCTATAACC	58	547	[40]
	CATI-R	TTGTAATTCATTAAGCATTCTGCC			
*cmlA*	cmlA-F	CCGCCACGGTGTTGTTGTTATC	58	698	
	cmlA-R	CACCTTGCCTGCCCATCATTAG			

#### 2.4.2. Virulence Factors

All *E. coli* strains were screened for the presence of 20 virulence genes associated with 7 distinct pathotypes: STEC, EHEC, EPEC, ETEC, EAEC, EIEC, and NTEC (Table 2). Specifically, the genes included *stx1* and *stx2*, which are characteristic markers of the STEC pathotype, along with the *hlyA* gene commonly found in EHEC strains. The *eaeA* gene shared by both EHEC and EPEC was also assessed. For EPEC, we targeted *escV*, *bfpB*, and *ent* genes; for ETEC, *elt*, *estIa*, and *estIb*; for EAEC, *astA*, *aggR*, and *pic*; for EIEC, *invE*; for NTEC, *cnf1*, *cnf2*, *cdt-I*, *cdt-II*, *cdt-III*, and *cdt-IV*.

**Table 2:** Primers and PCR conditions used for the detection of virulence genes.

Pathotype	Gene	Primer	Sequence 5′ → 3′	Annealing Temp. (°C)	Amplicon Size (bp)	References
STEC/ EHEC	*stx1*	stx1F	ATAAATCGCCATTCGTTGACTAC	60	180	[41]
		stx1R	GAACGCCCACTGAGATCATC			
	*stx2*	stx2F	GGCACTGTCTGAAACTGCTCC	60	255	
		stx2R	TCGCCAGTTATCTGACATTCTG			
	*hlyA*	hlyAF	GCATCATCAAGCGTACGTTCC	60	534	
		hlyAR	AATGAGCCAAGCTGGTTAAGCT			
EHEC/ EPEC	eaeA	eaeAF	GACCCGGCACAAGCATAAGC	60	384	
		eaeAR	CCACCTGCAGCAACAAGAGG			
EPEC	*escV*	MP3-escV-F	ATTCTGGCTCTCTTCTTCTTTATGGCTG	63	544	[42]
		MP3-escV-R	CGTCCCCTTTTACAAACTTCATCGC			
	*bfpB*	MP3-bfpB-F	GACACCTCATTGCTGAAGTCG	63	910	
		MP3-bfpB-R	CCAGAACACCTCCGTTATGC			
	*ent*	ent-F	TGGGCTAAAAGAAGACACACTG	63	629	
		ent-R	CAAGCATCCTGATTATCTCACC			
ETEC	*elt*	MP2-LT-F	GAACAGGAGGTTTCTGCGTTAGGTG	63	655	
		MP2-LT-R	CTTTCAATGGCTTTTTTTTGGGAGTC			
	*estIa*	MP4-STIa-F	CCTCTTTTAGYCAGACARCTGAATCASTTG	63	157	
		MP4-STIa-R	CAGGCAGGATTACAACAAAGTTCACAG			
	*estIb*	MP2-STI-F	TGTCTTTTTCACCTTTCGCTC	63	171	
		MP2-STI-R	CGGTACAAGCAGGATTACAACAC			
EIEC	*invE*	MP2-invE-F	CGATAGATGGCGAGAAATTATATCCCG	63	766	
		MP2-invE-R	CGATCAAGAATCCCTAACAGAAGAATCAC			
EAEC	*astA*	MP-astA-F	TGCCATCAACACAGTATATCCG	63	102	
		MP2-astA-R	ACGGCTTTGTAGTCCTTCCAT			
	*aggR*	MP2-aggR-F	ACGCAGAGTTGCCTGATAAAG	63	400	
		MP2-aggR-R	AATACAGAATCGTCAGCATCAGC			
	*pic*	MP2-pic-F	AGCCGTTTCCGCAGAAGCC	63	1111	
		MP2-pic-R	AAATGTCAGTGAACCGACGATTGG			
NTEC	*cnf1*	Cnf1F	GGGGGAAGTACAGAAGAATTA	55	1111	[43]
		Cnf1R	TTGCCGTCCACTCTCTCACCAGT			
	*cnf2*	Cnf2F	TATCATACGGCAGGAGGAAGCACC	55	1240	
		Cnf2R	GTCACAATAGACAATAATTTTCCG			
	*cdt-I*	Cdt1F	CAATAGTCGCCCACAGGA	56	412	
		Cdt1R	ATAATCAAGAACACCACCAC			
	*cdt-II*	Cdt2F	GAAAATAAATGGAATATAAATGTCCG	56	558	
		Cdt2R	TTTGTGTTGCCGCCGCTGGTGAAA			
	*cdt-III*	Cdt3F	GAAAATAAATGGAATATAAATGTCCG	56	558	
		Cdt3R	TTTGTGTCGGTGCAGCAGGGAAAA			
	*cdt-IV*	Cdt4F	CCTGATGGTTCAGGAGGCTGGTTC	56	350	
		Cdt4R	TTGCTCCAGAATCTATACCT			

Legend: EPEC: Enteropathogenic *Escherichia coli*, STEC: Shiga Toxin-producing *E. coli*, EHEC: Enterohemorrhagic *E. coli*, ETEC: Enterotoxigenic *E. coli*, EIEC: Enteroinvasive *E. coli*, EAEC: Enteroaggregative *E. coli*, NTEC: Necrotoxic *E. coli*.

## 3. Results

### 3.1. Escherichia coli Isolation

*Escherichia coli* strains were isolated from 54 (84.37%) out of 64 fecal samples examined. From each positive sample, a single *E. coli* isolate was selected for subsequent analyses. Confirmation of the strains as *E. coli* was achieved through PCR detection of the *usp*A gene. Table 3 summarizes the number of isolates obtained for each raptor species.

### 3.2. Antimicrobial Susceptibility Tests

Antimicrobial susceptibility testing was conducted on all 54 *E. coli* isolates, with the detailed results presented in Table 4. Most isolates demonstrated resistance to at least one antimicrobial agent. The highest resistance rates were observed for ampicillin, with 22/54 isolates (40.74%) resistant; enrofloxacin and ciprofloxacin, each with 17/54 (31.48%) resistant isolates; and tetracycline, with 14/54 isolates (25.93%) resistant.

Conversely, the highest susceptibility rates were noted for colistin, with all 54 isolates (100%) susceptible; followed by amikacin, with 51/54 isolates (94.44%) susceptible; ertapenem, with 49/54 isolates (90.74%) susceptible; gentamicin, with 47/54 isolates (87.04%) susceptible; chloramphenicol, with 46/54 isolates (85.19%) susceptible; and imipenem, aztreonam and trimethoprim-sulfamethoxazole, each with 44/54 isolates (81.48%) susceptible.

Based on the results of the antimicrobial susceptibility tests, the antimicrobial resistance profiles of the 54 isolates were characterized (Table 5 and Table 6). Of these, 16/54 (29.63%) isolates were classified as MDR, including 4 from buzzard, 3 from kestrel, 3 from peregrine falcon, 2 from tawny owl, 1 from little owl, 1 from barn owl, 1 from black kite, and 1 from sparrowhawk. Additionally, 4/54 (7.41%) isolates were identified as XDR, with one isolate each from a kestrel, a little owl, a peregrine falcon, and a barn owl. The remaining 34/54 (62.96%) isolates did not fall into any resistance category; among these, 24/54 (44.44%) isolates showed no resistance to any of the tested antimicrobials, whereas 10 (18.52%) exhibited resistance to at least 1 antimicrobial agent.

### 3.3. Genotypic Resistance

Molecular analyses were performed on 36/54 (66.67%) isolates that exhibited phenotypic resistance or intermediate susceptibility to penicillins and/or cephalosporins. Among these, 17/36 (47.22%) isolates tested positive for one or more resistance genes. Specifically, the *bla*_TEM_ gene was detected in 13/36 (36.11%) isolates, the *bla*_CTX-M_ gene in 4/36 (11.11%) isolates, the *bla*_CMY-2_ gene in 3/36 (8.33%) isolates, and the *bla*_SHV_ gene in 1/36 (2.78%) isolates.

The *bla*_CMY-1_ gene was not identified in any of the tested strains. Notably, some isolates harbored multiple resistance genes: one was positive for both *bla*_CMY2_ and *bla*_TEM_, another for *bla*_TEM_ and *bla*_CTX-M_, one for *bla*_SHV_ and *bla*_TEM_, and another for *bla*_CMY2_ and *bla*_CTX-M_.

Regarding carbapenem-resistant (5/54, 9.25%) or intermediate (8/54, 14.81%) isolates, none tested positive for carbapenemase genes *bla*_NDM_, *bla*_KPC_, *bla*_VIM_, *bla*_IMP_, or *bla*_OXA-48_.

In the case of tetracycline-resistant isolates, 13/14 (92.86%) carried *tet* genes. Specifically, 9/14 (64.29%) isolates harbored the *tet*(A) gene, and 4/14 (28.57%) isolates carried the *tet*(B) gene. No isolates harbored both genes simultaneously. The single isolate with intermediate susceptibility in the disk diffusion test was negative for both *tet*(A) and *tet*(B).

Among the eight chloramphenicol-resistant isolates (8/54, 14.81%), one carried the *cat1* gene, and two carried the *cmlA* gene; none exhibited both genes concurrently.

Table 5 and Table 6 summarize the resistance gene profiles of the tested *E. coli* isolates, while Table 7 reports the distribution of antimicrobial resistance genes across raptor species.

### 3.4. Virulence Factors

Among the virulence factors analyzed, only *ast*A, *eae*A, and *esc*V genes were detected (Table 5, Table 6 and Table 7). Notably, 15/54 (27.78%) isolates carried only the *ast*A gene. Additionally, 1/54 (1.85%) isolates tested positive for *ast*A, *eae*A, and *esc*V genes, and were classified as atypical EPEC (aEPEC).

Consequently, the following *E. coli* pathotypes were not identified in this study: STEC, EHEC, ETEC, EIEC, EAEC, and NTEC.

Table 5 and Table 6 summarize the virulent gene profiles of the tested *E. coli* isolates, while Table 7 reports the distribution of virulent genes across raptor species.

## 4. Discussion

Raptors are apex predators and mesopredators that forage across urban, rural, and natural environments, making them valuable bioindicators and sentinels of ecosystem health [44,45]. However, their scavenging behavior and position in the food chain also predispose them to accumulate pathogens and resistant bacteria, potentially turning them into reservoirs and vectors for the dissemination of these agents [23,26].

In the present study, *E. coli* isolated from raptors were characterized to assess their antimicrobial resistance and virulent traits. The examined birds showed no signs of intestinal or extraintestinal infection consistent with colibacillosis or other infectious disease; thus, they can be considered healthy from a clinical perspective. Consequently, the *E. coli* isolates obtained can be considered likely commensal bacteria, constituting part of the normal intestinal flora of their hosts. As previously reported, all samples were obtained from birds admitted to a rescue center. Fecal samples were collected promptly upon admission and prior to any medical treatment, ensuring that the center’s housing conditions and medical interventions did not influence the birds’ intestinal microflora. Therefore, it can be reasonably assumed that the bacterial strains recovered and analyzed most accurately represent the birds’ prior environmental exposures. In particular, the analyzed strains accurately represent the microbial exposure encountered by birds in their natural habitats. As such, they provide a comprehensive snapshot of ecosystem pollution, highlighting the presence of antimicrobial residues, resistant bacteria, and pathogenic organisms.

The initial focus of this study was to assess the antimicrobial resistance profile of the isolated *E. coli* strains. Notably, the highest resistance rates were observed against β-lactams, fluoroquinolones, and tetracycline.

For β-lactams, particularly, 40.74% of isolates exhibited resistance to ampicillin. Resistance to cephalosporins ranged from approximately 15 to 20%. Notably, only a few strains showed resistance to carbapenems. β-lactam antibiotics, including penicillins, cephalosporins, and carbapenems, remain cornerstone drugs in both human and veterinary medicine. However, their widespread overuse and misuse have contributed significantly to the proliferation of resistance [19,46]. Therefore, our results are not unexpected; in fact, with some exceptions, the resistance rates observed in our investigation are generally comparable to or lower than those reported in other studies involving raptors. For instance, only one study from the U.S.A. reported a lower percentage of β-lactams resistant *E. coli*, with a resistance rate of 12% to ampicillin in isolates from raptors [22]. Conversely, a research from Portugal on common buzzards found that 61.1% of *E. coli* isolates were resistant to ampicillin, and 44.4% showed resistance to cefoxitin [18]. Similarly, a study in Central Italy on wild birds reported that 85% of *E. coli* isolates were resistant to ampicillin, with about 10% resistant to other β-lactams tested; among the 59 strains from raptors, resistance levels were comparable [29]. Additionally, a study on black kites in southwestern Siberia found that 76% of *E. coli* isolates were resistant to ampicillin and 43% to cefotaxime [47].

Approximately 47.22% of the phenotypically β-lactams resistant *E. coli* tested positive for resistance genes. Notably, all genotypically positive strains exhibited resistance to at least ampicillin. The most frequently detected gene was *bla*_TEM_, identified in 36.11% of the resistant isolates. This finding aligns with the historical prominence of *bla*_TEM_ as the first ESBL gene to emerge and disseminate globally. Similar patterns have been observed in previous studies in *E. coli* isolates from raptors, which also reported a high prevalence of *bla*_TEM_ [18,22]. Other *bla* genes were detected at low frequencies or not at all in this study. This is consistent with their relative rarity, except for *bla*_CTX-M_, which has recently increased in prevalence. Currently, *bla*_CTX-M_ is considered the most widespread ESBL gene and a major contributor to β-lactams resistance worldwide [48].

The emergence of Extended Spectrum Beta-Lactamase (ESBL)-producing Enterobacteriaceae represents a growing global health concern, especially given the widespread use of beta-lactam antibiotics and the critical role of some as last-resort treatments. The relatively high detection rate of *bla* genes in wild raptors observed during this survey underscores the extensive dissemination of these resistance determinants across different environments. This finding highlights the potential for environmental pathways and ecological niches that may serve as reservoirs for these resistance genes and that can facilitate their spread.

Approximately 25% of the tested *E. coli* isolates exhibited resistance to tetracyclines. These findings align with the widespread use of these antimicrobials in animals, especially livestock, which has been shown to contribute significantly to the emergence and dissemination of resistance [49]. Consequently, our results are consistent with existing knowledge. However, the prevalence of tetracycline resistance in *E. coli* isolated from raptors varies considerably across different studies in the literature. For instance, a study conducted in Portugal focusing solely on common buzzards reported a high resistance rate of 75% [18]. In contrast, research from central Italy examining multiple raptor species documented a much lower resistance rate of approximately 8.47% [29]. Similarly, a study from the United States found that tetracycline was the most frequently detected resistance among *E. coli* from raptors, with about 16% isolates resistant [22]. These disparities likely reflect differences in geographic regions, level of anthropogenic influences, and the habitat preferences of the birds, particularly their exposure to human-related environments. Additionally, variation may be influenced by the specific raptor species examined, especially considering differences in their typical prey, which can impact the resistance gene profiles they acquire [50]. In our study, all but one of the tetracycline-resistant *E. coli* isolates tested positive for the targeted *tet* genes. Notably, we observed a higher positivity rate for *tet*(A) compared to *tet*(B). These two genes were selected based on their frequent detection in tetracycline-resistant *E. coli* [7], and our findings support this pattern. Limited literature is available regarding tetracycline resistance genes in *E. coli* from raptors. One study on common buzzards reported that 60% of tetracycline-resistant isolates harbored the *tet*(A) and/or *tet*(B) genes [18]. Another investigation in the U.S.A. identified *tet*(M) in tetracycline-resistant *E. coli*, albeit at a low detection rate [22].

A high proportion of strains tested in our study were not susceptible to fluoroquinolones, including enrofloxacin and ciprofloxacin. Quinolones are classified as “highest priority critically important antimicrobials (HPCIA)” by the World Health Organization due to their significance in human medicine [51]. However, some quinolones, particularly enrofloxacin, are widely used in veterinary medicine for both pets and livestock [52]. Given this context, our findings align with expectations, although fluctuations in resistance levels are evident across different studies. For example, a report from Italy documented 16% of ciprofloxacin-resistant *E. coli* in raptors [29], while a U.S.A. study reported 5.35% resistance in *E. coli* from raptors [22]. Additionally, research on black kites in southwestern Siberia found 13% ciprofloxacin-resistant strains [24]. Conversely, a study in Portugal reported that 50% of *E. coli* isolates from common buzzards were resistant to ciprofloxacin [18]. Resistance to these antimicrobials is generally associated with chromosomal mutations in genes coding for DNA gyrase and topoisomerase IV, rather than the presence of mobile resistance genes [7,24]. Consequently, the molecular mechanisms underlying resistance were not investigated in the present study.

A relatively small proportion (14.81%) of the tested isolates exhibited resistance to chloramphenicol. Excluding data from Portugal, where 41% of *E. coli* isolates from common buzzards were resistant [18], the overall resistance levels in *E. coli* form raptors remain low, generally under 10% [22,29]. This low prevalence is likely attributable to the ban on chloramphenicol use in livestock implemented in the mid-1990s in many countries [7]. Despite the relatively low number of chloramphenicol-resistant *E. coli* identified in our study, we conducted further investigations for transferable resistance genes, given the clinical significance of this antimicrobial in human medicine. Three of the isolates tested positive: one for the *cat1* gene and two for the *cmlA* gene. Similar findings have been reported by other researchers studying *E. coli* from raptors [18,22]. Both genes are commonly found in Enterobacteriaceae and confer chloramphenicol resistance through distinct mechanisms of action [7].

Regarding the other antimicrobials tested in this study, only a limited number of strains exhibited resistance to aminoglycosides and sulfonamides, while all strains were susceptible to colistin. Similar findings have been reported by other researchers studying *E. coli* isolated from raptors [18,22,29]. Given the growing concern over colistin resistance in recent years, particularly with the emergence and dissemination of mobile colistin resistance genes, these results are reassuring. They suggest a low or negligible impact of colistin resistance within the studied population and geographic area.

Considering the phenotypic and genotypic resistance data discussed above, some discrepancies have emerged. Specifically, certain strains exhibited phenotypic resistance to specific antimicrobials despite testing negative for all the resistance genes examined. Since *E. coli* is not intrinsically resistant to any antimicrobials, all observed resistance must be considered acquired [32]. The resistance genes selected for this study were chosen based on their clinical significance and epidemiological relevance, as they are among the most frequently detected in *E. coli* and other Enterobacteriaceae. Nonetheless, other resistance mechanisms have been described in this species, including chromosomal mutations and less commonly identified mobile resistance genes, which may account for some of the phenotypic resistance observed without corresponding genotypic markers [7].

Based on antimicrobial resistance results, we were able to assess the multidrug resistance profiles of the isolated *E. coli* strains. Co-resistance to antimicrobials across different classes significantly limits therapeutic options, posing a major challenge in infectious disease management. Consequently, this topic holds considerable importance and relevance. In our study, although 44.44% of the tested strains did not exhibit resistance to any of the evaluated antimicrobials, 37.04% were multidrug-resistant. Specifically, 29.63% were classified as MDR, and 7.41% as XDR. Data on multidrug resistance in *E. coli* from raptors are limited and show considerable variability across different studies. For example, research conducted in southwestern Siberia on *E. coli* isolated from black kites reported a high MDR prevalence of 94.1% [24]. Similarly, a study on *E. coli* from common buzzards in Portugal, although not specifying the exact number of MDR strains, noted that only one out of 36 isolates was susceptible to all tested antimicrobials [18]. Conversely, a study from the U.S.A. found that just 8% *E. coli* isolates from raptors exhibited MDR profiles [22]. In Central Italy, one study reported less than 20% MDR *E. coli* [29], while another from northern Italy observed a prevalence of 39.6% [19]. These variations may be influenced by differences in raptor species, geographic locations, and environmental factors. As apex predators, birds of prey can serve as valuable bioindicators, reflecting the presence and circulation of antimicrobial-resistant bacteria within specific habitats. These resistance patterns are likely shaped by multiple factors, particularly human activity and environmental contamination.

The second focus of this study was to assess the carriage of pathogenic *E. coli* by raptors. The findings suggest that raptors play a limited role in the dissemination of the enteric *E. coli* pathotypes. Specifically, none of the tested isolates belonged to STEC, EHEC, ETEC, EIEC, EAEC, or NTEC; only one aEPEC strain was identified, and 27.78% of isolates only carried the *ast*A gene. Although *E. coli* strains from all these pathogenic groups are significant human health concerns, data on their presence in raptors remain scarce. Existing studies often do not distinguish between wild bird orders or species, especially those involving older or negative results. Generally, wild birds can serve as carriers of pathogenic *E. coli*, but detection rates tend to be low. Non-raptor birds, such as Passeriformes, Anseriformes, or Columbiformes, are more frequently found positive for InPEC [27,53,54]. Regarding raptors, a Brazilian study reported the isolation of one *eae* positive *E. coli* from 37 birds of the order Strigiformes, all of which were negative for *stx* genes [54]. Similarly, a Spanish study detected *eae*-positive *E. coli* in one owl among 79 strains from various bird species [55]. In southwestern Siberia, in 36 *E. coli* isolates from black kites, researchers revealed the presence of the *cnf1* gene in one strain [24]. Additionally, a previous culture-independent study carried out in the same geographic area of the present investigation identified *stx1*, *stx2*, *eaeA,* and *hylA* genes in the intestine of one little owl out of nine tested raptors across different species [27]. Overall, our findings confirm that diurnal and nocturnal raptors have a minimal role as carriers, reservoirs, and spreaders of InPEC, despite being apex predators. However, the detection of virulent strains warrants attention, as it indicates the circulation of *E. coli* harboring selected virulence determinants in the area studied, highlighting the potential of raptors as bioindicators. Moreover, these strains pose risks of severe illnesses to both humans and domestic animals. Although atypical EPEC are generally less virulent than typical EPEC (tEPEC), they can still cause disease [2]. While tEPEC have primarily humans as reservoirs, aEPEC are zoonotic, maintained and transmitted to humans by animals [4]. Our data underscore the presence of aEPEC among wild bird populations and extend knowledge about potential carrier species. Notably, 27.78% of isolates carried the *ast*A gene, which encodes a heat-stable toxin (EAST1) associated with secretory diarrhea. While commonly linked to EAEC, *ast*A is also found in other pathotypes such as ETEC and DAEC [2]. Furthermore, *astA* can be located either on the chromosome or on plasmids, which enhances its ability to spread efficiently between isolates. Although EAST1 shares structural similarities with the heat-stable enterotoxin encoded by ETEC, its individual functional significance remains uncertain in the absence of other virulence factors. It is likely that this toxin plays a supportive role, enhancing the activity of other virulent determinants rather than acting as a primary virulence factor on its own [2]. However, the sole presence of *ast*A, without other pathogenic markers, is sufficient to cause diarrhea in humans [56]. Our results reveal an active circulation of these virulent *E. coli* strains within the studied area and among the sampled bird population. They also expand our understanding of the potential spreaders of *E. coli* harboring selected virulence determinants, emphasizing the importance of monitoring wildlife as indicators of environmental, veterinary, and public health risks, in the One Health perspective.

The present study has some limitations that should be acknowledged. Firstly, the sample size of animals included was relatively limited. While working with wildlife, particularly non-hunting and protected species, poses significant challenges, increasing the number of raptors studied would enhance the representativeness and robustness of the findings. Additionally, for some species, only one or a few individuals were sampled, which restricts the ability to assess species-specific contributions to the observed results.

The second limitation pertains to the opportunistic sampling strategy employed. Specifically, only raptors admitted to a wildlife rescue center, primarily due to trauma, were included. Although this approach is ethically advantageous, avoiding the trapping and stress associated with active capture, it may not yield a sample that accurately reflects the broader wild population, potentially introducing bias.

Finally, we limited our analysis to a single colony from each sample to optimize resource use and enable more extensive analyses. However, expanding the sampling to include multiple colonies from the same animal would provide a broader perspective on the within-host diversity of *E. coli*, thereby enriching our understanding of its ecological and epidemiological dynamics.

## 5. Conclusions

The present study reinforces evidence that raptors can harbor *E. coli* exhibiting relevant antimicrobial resistance phenotypes, resistance genes, and selected virulence determinants. The resistance patterns observed were predominantly associated with resistance to aminopenicillins, fluoroquinolones, and tetracyclines. The detection of some mobile resistance genes further corroborates the potential of raptors to serve as reservoirs of clinically significant resistance traits.

Additionally, the identification of virulent *E. coli*, such as *astA*-positive isolates and atypical EPEC, emphasizes the capacity of these birds to harbor potentially zoonotic strains.

The coexistence of resistance traits and virulence genes within certain isolates raises concerns regarding their pathogenic potential and the risk of transmission to domestic animals or humans.

Our findings confirm that raptors are effective bioindicators for antimicrobial resistance and virulent bacteria in the environment, reflecting exposure to anthropogenic, livestock, and natural reservoirs within their foraging territories. Their ecological characteristics, including mobility and frequent interaction with human-related habitats, make them not only sensitive bioindicators of ecosystem health but also potential vectors of resistant bacteria.

Lastly, this research highlights the dual role of wildlife rescue centers as both healthcare facilities for injured wildlife and critical monitoring points for environmental health. It underscores the need for strict biosecurity protocols within these facilities to prevent the spread of antimicrobial-resistant and virulent bacteria, and to protect the health and safety of center personnel.

## Figures and Tables

**Table 3:** Sampled birds and the number of isolated *Escherichia coli* strains.

Common Name	Scientific Name	Number of Tested Animals	Isolated *E. coli*
Eurasian buzzard	*Buteo buteo*	13	12
Common kestrel	*Falco tinnunculus*	10	10
Little owl	*Athene noctua*	10	9
Peregrine falcon	*Falco peregrinus*	9	7
Barn owl	*Tyto alba*	7	5
Tawny owl	*Strix aluco*	7	5
Scops owl	*Otus scops*	3	1
Black kite	*Milvus migrans*	2	2
Sparrowhawk	*Accipiter nisus*	2	2
Honey buzzard	*Pernis apivorus*	1	1
Total		64	54

**Table 4:** Results of the antimicrobial susceptibility tests.

Antimicrobials		Resistant		Intermediate		Susceptible	
Class	Antimicrobial	Number of Isolates	%	Number of Isolates	%	Number of Isolates	%
Penicillins	Ampicillin	22	40.74	7	12.96	25	46.30
	Amoxicillin-clavulanate	8	14.81	11	20.37	35	64.81
Cephalosporins	Cefoxitin	11	20.37	4	7.41	39	72.22
	Cefotaxime	9	16.67	12	22.22	33	61.11
	Ceftiofur	8	14.81	5	9.26	41	75.93
Carbapenems	Imipenem	3	5.56	7	12.96	44	81.48
	Ertapenem	4	7.41	1	1.85	49	90.74
Monobactams	Aztreonam	8	14.81	2	3.70	44	81.48
Phenicols	Chloramphenicol	8	14.81	0	0.00	46	85.19
Tetracyclines	Tetracycline	14	25.93	1	1.85	39	72.22
Fluoroquinolones	Enrofloxacin	17	31.48	5	9.26	32	59.26
	Ciprofloxacin	17	31.48	2	3.70	35	64.81
Aminoglycosides	Gentamicin	7	12.96	0	0.00	47	87.04
	Amikacin	1	1.85	2	3.70	51	94.44
Sulfonamides	Trimethoprim-sulfamethoxazole	10	18.52	0	0.00	44	81.48
Polymyxins	Colistin	0	0.00	0	0.00	54	100.00

**Table 5:** Phenotypic and genotypic resistance and virulence profiles of the non-multidrug-resistant isolates.

Strain Number	Bird Species (Number of Isolates)	Antimicrobial Resistance Profile	Resistance Genes	Virulence Genes
15, 16, 20, 21, 59, 125, 144, 145, 146, 150, 157, 158, 160, 161	Peregrine falcon (3) Eurasian buzzard (2) Common kestrel (1) Little owl (2) Barn owl (2) Scops owl (1) Tawny owl (2) Black kite (1)	not resistant to all molecules		
14, 24, 25, 26, 34, 90, 126, 136, 159	Eurasian buzzard (4) Common kestrel (3) Little owl (2)	not resistant to all molecules		*ast*A
127	Eurasian buzzard (1)	not resistant to all molecules		*ast*A, *eae*A, *esc*V
17	Eurasian buzzard (1)	SXT		*ast*A
18	Little owl (1)	SXT		*ast*A
23	Little owl (1)	AMP, AMC, FOX		
27	Little owl (1)	ATM		
58	Common kestrel (1)	AMP	*bla*_TEM_	
143	Barn owl (1)	AMP		
147	Tawny owl (1)	IPM, ETP		*ast*A
153	Honey buzzard (1)	FOX		
163	Common kestrel (1)	TE	*tet*(A)	*ast*A
164	Sparrow hawk (1)	TE	*tet*(A)	

Legend: AMP: ampicillin; AMC: amoxicillin-clavulanate; ATM: aztreonam; FOX: cefotaxime; SXT: trimethoprim-sulfamethoxazole; TE: tetracycline.

**Table 6:** Phenotypic and genotypic resistance and virulence profiles of the MDR and XDR isolates.

Strain Number	Bird Species (Number of Isolates)	Antimicrobial Resistance Profile	Resistance Genes	Virulence Genes
22	Little owl (1)	AMP, AMC, FOX, ENR		
30	Eurasian buzzard (1)	AMP, TE, ENR, CIP, SXT	*bla*_TEM_, *tet*(B)	*ast*A
45	Eurasian buzzard (1)	AMP, C, TE, ENR, CIP, SXT	*bla*_TEM_, *tet*(A), *cmIa*	
46	Peregrine falcon (1)	AMP, C, TE, ENR, CIP	*bla*_TEM_, *tet*(A), *cmIa*	
47	Peregrine falcon (1)	C, TE, ENR, CIP, CN, SXT	*tet*(B)	
137	Common kestrel (1)	AMP, AMC, FOX, ENR, CIP	*bla*_CMY2_	
138	Common kestrel (1)	AMP, TE, ENR, CIP	*bla*_TEM_	
142	Eurasian buzzard (1)	AMP, AMC, FOX, CTX, TE, ENR, CIP	*bla*_CMY2_, *bla*_TEM_, *tet*(A)	
148	Tawny owl (1)	AMP, C, TE	*bla*_TEM_, *tet*(A)	
149	Tawny owl (1)	AMP, AMC, FOX, CTX, FUR, IPM, ETP, ATM, CIP		
151	Black kite (1)	AMP, CTX, FUR, ATM, ENR, CIP, CN	*bla*_CTX-M_	
152	Sparrowhawk (1)	AMP, FOX, FUR, C, ENR, CIP	*bla*_TEM_	
154	Common kestrel (1)	AMP, FOX, IPM, TE, ENR, CIP, SXT	*bla*_TEM_, *bla*_CTX-M_, *tet*(A)	*ast*A
155	Eurasian buzzard (1)	AMP, CTX, FUR, ETP, ATM, ENR, CIP, CN		
156	Barn owl (1)	AMP, AMC, CTX, SXT	*bla*_TEM_	
162	Peregrine falcon (1)	AMP, ENR, CIP, CN	*bla*_TEM_	
31	Barn owl (1)	AMP, CTX, FUR, ATM, C, TE, ENR, CIP, SXT	*bla*_SHV_, *bla*_TEM_, *tet*(B), *cat*1	
139	Common kestrel (1)	AMP, FOX, CTX, FUR, ETP, ATM, TE, ENR, CIP, CN, AK	*bla*_TEM_, *tet*(B)	
140	Peregrine falcon (1)	AMP, AMC, FOX, CTX, FUR, ATM, C, TE, ENR, CIP, CN, SXT	*bla*_CMY2_, *bla*_CTX-M_, *tet*(A)	
141	Little owl (1)	AMP, AMC, FOX, CTX, FUR, ATM, C, TE, ENR, CIP, CN, SXT	*bla*_CTX-M_, *tet*(A)	

Legend: AK: amikacin; AMP: ampicillin; AMC: amoxicillin-clavulanate; ATM: aztreonam; C: chloramphenicol; CIP: ciprofloxacin; CN: gentamicin; CTX: cefotaxime; ENR: enrofloxacin; ETP: ertapenem; FOX: cefotaxime; FUR: ceftiofur; SXT: trimethoprim-sulfamethoxazole; TE: tetracycline; Light blue = MDR strains; light green = XDR strains.

**Table 7:** Distribution of virulence and resistance genes in relation to raptor species.

Species	Analyzed Strains	Virulence Genes			Resistance Genes							
		*eaeA*	*escV*	*ast*A	*bla*_CMY2_	*bla*_SHV_	*bla*_CTX-M_	*bla*_TEM_	*tet*(A)	*tet*(B)	*cat*1	*cmIa*
Eurasian buzzard	12	1	1	7	1	0	0	3	2	1	0	1
Common kestrel	10	0	0	5	1	0	1	4	2	1	0	0
Little owl	9	0	0	3	0	0	1	0	1	0	0	0
Peregrine falcon	7	0	0	0	1	0	1	2	2	1	0	1
Barn owl	5	0	0	0	0	1	0	2	0	1	1	0
Tawny owl	5	0	0	1	0	0	0	1	1	0	0	0
Scops owl	1	0	0	0	0	0	0	0	0	0	0	0
Black kite	2	0	0	0	0	0	1	0	0	0	0	0
Sparrowhawk	2	0	0	0	0	0	0	1	1	0	0	0
Honey buzzard	1	0	0	0	0	0	0	0	0	0	0	0
**Total**	**54**	**1**	**1**	**16**	**3**	**1**	**4**	**13**	**9**	**4**	**1**	**2**

## Data Availability

The original contributions presented in this study are included in the article. Further inquiries can be directed to the corresponding author.
